# Subjective (dis)utility of effort: mentally and physically demanding tasks

**DOI:** 10.1186/s41235-020-00226-5

**Published:** 2020-06-05

**Authors:** Phillip L. Ackerman, Corey E. Tatel, Sibley F. Lyndgaard

**Affiliations:** School of Psychology, Georgia Institute of Technology, 654 Cherry Street, MC 0170, Atlanta, GA 30332-0170 USA

## Abstract

Effort as a concept, whether momentary, sustained, or as a function of different task conditions, is of critical importance to resource theories of attention, fatigue/boredom, workplace motivation, career selection, performance, job incentives, and other applied psychology concerns. Various models of motivation suggest that there is an inverted-U-shaped function describing the personal utility of effort, but there are expected to be individual differences in the optimal levels of effort that also are related to specific domain preferences. The current study assessed the disutility of effort for 125 different tasks/activities and also explored individual differences correlates of task preferences, in a sample of 77 undergraduate participants. The participants rated each activity in terms of the amount of compensation they would require to perform the task for a period of 4 h. They also completed paired comparisons for a subset of 24 items, followed by a set of preference judgments. Multidimensional scaling and preference scaling techniques were used to determine individual differences in task preference. Personality, motivation, and interest traits were shown to be substantially related to task preferences. Implications for understanding which individuals are oriented toward or away from tasks with different effort demands are discussed, along with considerations for the dynamics of attentional effort allocations during task performance.

## Significance

A fundamental problem of applied psychology relates to generalizing laboratory-based studies of attention and effort to real-world situations, such as classroom learning or job performance—essentially an issue of the ecological validity of laboratory research (Brunswik, 1943). Although researchers often obtain high levels of effort from study participants in the laboratory when there is a direct compensation of course credit or monetary rewards (or the researchers discard the data from participants who do not maintain an acceptable level of effort during the study), effort fluctuations are often pronounced when individuals are in a classroom or reading for homework. The current study attempts to bridge this gap by examining the underlying subjective perceptions of effortful tasks, and exploring individual differences in task preferences, as a function of select personality and interest traits. From the results, we confirm the hypothesis of motivation theorists that average subjective preferences have an inverted-U-shaped function with level of mental effort demanded by tasks. In addition, there are substantial individual differences in the types of tasks that are viewed as aversive, which in turn are correlated with personality and interest variables. An important implication of this work is that conflicting results of the effects of task performance over extended time-on-task (e.g., fatigue, boredom, vigilance) may be at least partially resolved by attention to individual and group differences in the subjective disutility of effort on different tasks.

## Introduction

Assessment of an individual’s “subjective” effort for mentally demanding tasks is a difficult enterprise, because there are no objective physical manifestations of the engagement of an individual in the task, and there are likely individual differences in the perception of work/effort expended in an activity, depending partly on whether the individual enjoys or wishes to avoid the task (e.g., reading for pleasure vs. reading an assigned textbook for a test—see Dodge, 1913 for a discussion). Moreover, individual differences in perceived effort across different tasks represent a source of substantial variability in studies where researchers might hope to have uniform demands on research participants.

“Effort” is a singularly important construct in the study of attention (e.g., Kahneman, 1973), and the perception of expended effort is also integral to theories of cognitive fatigue and other applications in the area of ergonomics/human factors (e.g., for a review, see Ackerman, 2011). In addition, the subjective disutility of effort is also a key ingredient to theories of personality and motivation, along with aspects of vocational choice. Because little is known about how adults view a wide array of tasks and activities in terms of disutility, we designed the current study to explore the perceptual judgments that determine the disutility and attractiveness/aversiveness of tasks. The goals of the study were as follows: (1) Explore the mean subjective disutility for a variety of mentally demanding and physically demanding tasks/activities; (2) determine whether the subjective Effort-Utility function conforms to an inverted-U shape; (3) evaluate the dimensionality of perceptual space of tasks/activities to better understand how subjective disutility judgments are made and to evaluate the similarities and differences among perceptions of different tasks/activities; (4) determine whether individual differences in effort preferences within the multidimensional task/activity space are best represented by vector (more is better) or ideal point models; and (5) evaluate whether individual differences in key personality and motivational traits are related to differences in the subjective disutility of effort and task preferences.

## Background

### Effort as a disutility

Experimental work on behavioral decision making suggests that, *ceteris paribus*, people tend to prefer less cognitively demanding tasks (e.g., Kool, McGuire, Rosen, & Botvinick, 2010). The cognitive effort discounting paradigm (COGED) is a model that quantifies the cost of using this limited resource (Westbrook & Braver, 2015; Westbrook, Kester, & Braver, 2013). The COGED paradigm was developed through a series of choice experiments that asked participants whether they would prefer to complete a more demanding task for more money or a less demanding task for less money. By identifying a point of “indifference” between the levels of tasks, Westbrook and colleagues were able to quantify individuals’ subjective value of engaging in effortful tasks by calculating the difference between the monetary rewards associated with the more demanding task and the less engaging task.

Evidence for the subjective value of expending physical effort suggests that there is a parabolic relationship between effort and subjective disutility such that as physical effort increases from 0 to 100% of capability, subjective disutility increases (Hartmann, Hager, Hartmann, Hager, Tobler, & Kaiser, 2013). Hartmann and colleagues found that increased physical effort (intensity of squeezing a handgrip) was associated with higher subjective disutility. However, that study was unidimensional, in that it did not provide evidence for differing types of physical tasks. It could be that tasks similar in physical effort result in varying levels of subjective utility if they differ on other domains (i.e., attractiveness-aversiveness). The authors also did not measure individual trait differences that may have influenced perceptions of disutility.

The study of physically demanding tasks has been extended to consider cognitive tasks. For example, Kool and colleagues (2010) used demand selection tasks to determine that people generally prefer tasks that require lower levels of effort, which provides evidence that people are biased toward tasks that require minimal mental effort. However, similar to the Hartmann et al. (2013) investigation of physical tasks, the task paradigms did not differ with respect to the content of mental effort required (e.g., numerical, verbal, spatial) or with respect to how attractive/aversive the tasks were. Given that a person’s verbal and visual-spatial working memory capacities have been linked to strategy selection on tasks that require mental effort (MacLeod, Hunt, & Mathews, 1978), it is possible that people are more inclined to engage in effortful tasks that align best with their perceived abilities.

### A more nuanced relationship between effort and perceived task disutility

While the perspectives reviewed in the previous section suggest a ubiquitous aversion to expending physical and mental effort, this narrative is relatively limited. The purpose of the current study was to investigate a more nuanced conceptualization of the perceived disutility of effort. In this section, we review theoretical frameworks and empirical evidence from multiple fields of psychology that inform our view that the relationship between the amount of effort that tasks require and their disutility is not as simple as traditional perspectives suggest.

#### Attention theory

Although effort is a central focus of Kahneman’s (1973) theory, the level of effort a participant allocates to a task is largely attributable to only experimental conditions (such as task difficulty) and capacity changes associated with changes in arousal. Issues of volition (in terms of marginal changes in rewards for correct performance) were deemed to have a “marginal” effect on arousal and thus effort allocated to a task. Kahneman argued that “the subject simply cannot try as hard in a relatively easy task as he[/she] does when the task becomes more demanding.” (p. 14). Other effort models have taken a more nuanced approach to fluctuations of attention availability in response to stress or boredom (e.g., Hockey, 1993), but in these various frameworks, there is no role for motivation or individual differences in task preferences as inputs for attentional allocations to tasks. Nonetheless, there have been suggestions in the literature that dynamic changes in effort allocations are instrumental in effects as varied as the vigilance decrement (e.g., Dember, Galinsky, & Warm, 1992) and fatigue (Hockey, 2011), but also related to memory in general (for a review, see Mitchell & Hunt, 1989). Effort allocations may be determined to a degree by imposed task conditions, but it is possible that individual differences in approach/aversion to different tasks may also play an important role in determining effort. Three individual differences domains (motivation, personality, and interests) relevant to the current study are discussed in the following sections.

#### Motivational theories

As a contrast, in motivational theories such as Kanfer’s (1987) formulation that uses Kahneman’s model as the starting point, the amount of effort an individual devotes to a job or task is a result of three different functions: the subjective Effort-Utility function; the Performance-Utility function, which describes the relationship between the level of job/task performance and the utility of rewards, such as wages; and the Effort-Performance function (see Norman & Bobrow, 1975), which describes the relationship between the amount of effort needed for different levels of task performance. The Performance-Utility function is largely related to external contingencies, such as goals or performance standards. The Effort-Performance function is determined by task characteristics (e.g., attentional demands, task difficulty). An individual’s decision to expend a particular level of effort on the job is expected to result from a calculation between the individual’s desired level of effort, the relationship between different levels of effort and resulting levels of performance, and the punishment/rewards associated with different levels of performance.

A variety of achievement motivation theories and empirical research (e.g., Atkinson & Feather, 1966; for a review, see Kanfer, 1990) suggest that individuals who are high in need for Achievement (nAch) tend to prefer tasks that have a “moderate” level of difficulty (and consequently, a relatively high likelihood of performance success). The implication of the achievement motivation framework is that the general form of the subjective Effort-Utility function is an inverted U (Kanfer, 1987; see also McGrath, 1976). That is, tasks with too-little effort demands (boredom) and tasks with too-high effort demands (fatigue) are less desirable/more aversive than tasks with moderate effort demands. Most critically, the inference is that these functions may differ between individuals or developmentally within individuals (e.g., with aging).

#### Personality

One area of inquiry relevant to understanding individual differences in the subjective disutility of mental effort in particular is associated with the personality trait called by various names, such as Intellect (Digman & Takemoto-Chock, 1981), need for Cognition (Cacioppo & Petty, 1982), and Typical Intellectual Engagement (Goff & Ackerman, 1992). Although approaches to these constructs are not identical, they tend to share a conceptualization that there is a relatively stable trait along which people differ, that relates to an orientation toward or away from expenditures of mental (or intellectual) effort, also known as “investment” (see von Stumm & Ackerman, 2013, for a review and meta-analysis). People who are high on these traits express an interest in performing intellectually demanding tasks (e.g., reading, abstract thinking, problem solving), while those low on these traits express a desire to avoid such activities. Related variables from the motivational trait and skill domain include traits of Mastery and Desire to Learn (e.g., Kanfer & Heggestad, 1997; Spence & Helmreich, 1983).

#### Interests

Numerous investigations in the realm of vocational interest assessments and career counseling concern themselves with assessing the subjective attractiveness or aversion to different kinds of jobs and leisure activities. Traditionally, such assessments take a variety of different forms, from ratings of like/dislike of specific jobs/activities to contrasts between pairs of jobs/activities (e.g., see Holland, 1997a; Strong Jr., 1943). These assessments are generally designed to determine an individual’s dominant occupational theme or themes (in the case of the Holland model) or to evaluate the similarity of an individual’s interests to those of job incumbents (e.g., Strong’s model).

The dominant framework for vocational interests (Holland, 1997a) has suggested that there are six major themes of interests (Realistic, Investigative, Artistic, Social, Enterprising, and Conventional) that correspond to various families of occupations/jobs. One shortcoming of the Holland approach, however, is that even though there are correlations between the six occupational themes and intellectual abilities, the scales are largely designed to be independent of the mental/physical effort demands of particular occupations within each of the themes (e.g., for a discussion, see Toker & Ackerman, 2012). For example, two individuals might have nearly identical dominant vocational interest themes, but one individual may desire to be a neurosurgeon, and the other, a medical lab technician—two occupations which presumably differ substantially in terms of mental effort demands, and these are not explicitly distinguished in the standard implementation of Holland’s model. Moreover, even two different lab technicians with the same interest profiles may differ substantially in terms of how much effort they wish to bring to the job on a daily basis. One may simply do the minimum required for the job, while the other has a high degree of dedication and exerts maximum effort most days.

Thus, what is missing from these approaches is a sense of either the average or the nature of individual differences in the subjective disutility of effort. An individual with a lower disutility for high levels of effort should be expected to gravitate to jobs/tasks that are more demanding, while an individual with a higher disutility for high levels of effort should gravitate to jobs/tasks that are comparatively less demanding. We argue, consistent with Kool and colleagues (2010), that individual differences which make some people more or less averse to expending cognitive effort do not negate an overarching “law of less mental effort” (Kool et al., 2010). Rather, understanding this subjective disutility of effort may help to better explain the circumstances under which some people are more willing to exert effort.

### Measurement of the attractiveness/aversiveness of tasks

Different fields of psychology either implicitly or explicitly attempt to determine both the mean attractiveness/aversiveness of tasks and the nature of individual differences in preferences. The explicit approaches often center on vocational interests (e.g., Holland, 1997a; Strong Jr., 1943). Individuals are asked about whether they would “like” or “dislike” particular jobs and tasks, with the goal of determining the kinds of jobs with which individuals would be satisfied or dissatisfied, in terms of educational and occupational placement. Implicit approaches to estimating attractiveness of tasks even pervade the conduct of psychological experiments. Researchers typically expect that experiments consisting of more aversive tasks will require higher compensation in order to recruit a sample of sufficient size. In addition, there are within-study considerations. Tasks that require greater levels of mental or physical effort over extended periods of time often result in changes to the individual’s level of effort over time (e.g., fatigue), while tasks that “engage” the participants may show stable or increasing levels of expended task effort over time (e.g., see Ackerman, 2011 for a review). Video games, for example, are notable for the degree of intrinsic interest they generate, especially among adolescents and young adults, which often leads to voluntary engagement for many hours without interruption (e.g., Malone, 1981).

Nonetheless, there are relatively few empirical studies in this domain that allow one to directly estimate the subjective disutility of different kinds of mentally and physically demanding tasks. Thorndike (1937) was perhaps the first psychologist to explore the disutilities individuals have for different kinds of situations. He asked participants to indicate how much money they would require to suffer one of several different discomforts or deprivations (e.g., “Eat a live beetle one inch long” or “Have to live the rest of your life in New York City”). Even though Thorndike did not investigate any tasks or job-type activities as stimulus variables, his was a useful approach to understanding attitudes toward the relative aversiveness of different kinds of phenomena. Given that a person’s subjective perception of effort has implications for a variety of concerns related to career selection and organizational behavior, the purpose of the current study was to explore the disutility of effort for tasks that differ in regard to physical and mental characteristics.

While physical effort can be objectively estimated in terms of gross and fine motor activities and calories expended, there is no analogous objective measure for mental effort. Thus, it is necessary to obtain subjective ratings of effort to assess the costs or disutilities of tasks requiring mental effort. Because previous researchers (e.g., Dodge, 1913; Thorndike, 1937) have identified the underlying diversity of subjective disutilities for particular tasks (e.g., two individuals may have extremely different levels of aversion to a particular task), determining the nature of subjective judgments of disutility requires an approach that samples widely among various task types and demands. We therefore created a large set of tasks that included those that involved different abilities or cognitive resource pools (Wickens, 1980), such as encoding, processing, responding; and spatial, verbal, numerical, and perceptual content, and we sampled tasks that are often seen as more or less intrinsically interesting, at least to some individuals.

## The current study

### Overview

To accomplish these goals, we created a set of 125 different tasks/activities as stimulus items (for simplicity, they are referred to as “tasks” in the rest of this paper). Measures of personality, motivation, and related traits were assessed first, in a set of questionnaires completed at home. In a single laboratory session, participants rated the subjective disutility of the tasks in terms of the amount of money per hour they would require to perform the task over 4 h. A subset of the items was then administered in a paired-comparison format, to be used as input for multidimensional scaling (MDS) of the perceptual space for task effort demands. Finally, preference judgments were obtained for the subset of stimuli and then subjected to preference scaling in the context of the MDS of similarity judgment data—using an approach similar to that of O’Hare’s (1976) study of perceived similarity and individual differences for preference of visual art. Results are provided in terms of exploring the perceptual space of effort disutility, task similarity, preferences, and trait correlates of task preferences.

### Hypotheses

Because extant theories of motivation (e.g., Kanfer, 1987) propose that people generally are attracted to tasks with a “moderate” level of effort, and avoid tasks of both extremely low demands (boredom) and extremely high effort demands (fatigue), we hypothesized that the function relating task disutility to effort would have an inverted-U shape.

- *H1. A quadratic function will provide a significant and parsimonious fit to the regression of effort demands on rated task disutility.*

The underlying perceptual structure of effort is best represented by a multidimensional structure when tasks are sampled across content domains (e.g., physical, cognitive [verbal, spatial, numerical]). That is, we expected to find that perceived effort is not a unitary construct, but is differentiated.

- *H2. A multiple dimensional structure will provide a more parsimonious fit than a single dimension to the judged similarities of a sample of different tasks.*

We expected that individuals would show marked differences in the nature of preferred tasks or tasks to be avoided and that preference models would reveal these differences. These differences were expected to be characterized by differential preference solutions across participants (e.g., vector model, ideal point model). A vector model describes an individual who prefers “more” of a particular quality, beyond the limit of the stimuli judged. An example of a vector model would be the amount of money a person would like to win in a lottery. Winning $50 would be perceived as more preferable to receiving $2, receiving $100 would be more preferable to either, and so on. Ideal point models (also known as “unfolding” models) indicate that there is a “sweet spot” for the individual’s preferences. Levels of the dimension that are more or less than the ideal point are less desirable. The classic example for ideal–point models is the temperature and amount of sugar preferred in a cup of tea (e.g., see Carroll, 1972). A cup of tea that is colder or hotter than the ideal, or that contains more or less sugar than the ideal, is judged to be a less preferable alternative.

- *H3. Some individuals will be best described by a vector model (where “more” along the task dimension is most desirable), while others may be best described by an ideal point model (where the most desirable task would have characteristics within the range of sampled tasks).*

Finally, it was anticipated that self-concept, interests, and salient constellations of traits (trait complexes^1^) would be related to the pattern of preferences. Individuals who perceive higher levels of verbal abilities were expected to have lower levels of disutility for tasks that involve reading or writing, those who perceive higher levels of math abilities were expected to have relatively lower levels of disutility for tasks that involve math or spatial content, and those who have higher levels of “facilitative” personality and interest traits (e.g., Mastery, need for Achievement, Openness) were expected to have lower levels of disutility for cognitively demanding tasks, compared to individuals who have lower levels on such traits.

- *H4. Trait measures will be substantially correlated with task preferences.*

## Method

### Participants

Eighty-five individuals participated in the study. Participants were undergraduate students, over the age of 18 and fluent speakers/readers of English, who were compensated with course research credit. Seven participants did not complete all phases of the study, and their data were not considered. One participant did not follow instructions, and that participant’s data were also deleted. Complete data were obtained from 77 participants (44 men and 33 women).

### Measures

There were four components to the study: (1) at-home questionnaire, (2) disutility judgments, (3) similarity judgments, and (4) preference judgments. Each component is described below, in the order in which they were completed by participants.

#### At-home questionnaire

The questionnaire included self-report assessments of several self-concepts, self-estimate of ability, personality, motivation, and interest traits that were hypothesized to be related to individual differences in preferences for different kinds of mental and physical activities, along with a sample set of self-efficacy questions related to five of the activities from the disutility, similarity, and preference items. The specific measures used are described in the following paragraphs.

##### 4.2.1.1. Self-concept and self-estimates of ability

Measures of self-concept contained 18 statements about verbal, math, and spatial abilities and skills. Participants rated whether they agreed or disagreed with statements about their abilities and skills (response scale: 1 = Strongly Disagree to 6 = Strongly Agree). In addition, an 11-item measure of Preference for Numerical Information (Viswanathan, 1993) was included to assess related aspects of math self-concept and attitudes. Measures of self-estimates of ability included a range of different abilities, including Verbal, Math, Spatial, Perceptual Speed/Psychomotor, and General. The instructions indicated that participants were to choose a percentile rank for each indicating how they compared with others (response scale: [1 = Extremely Low to 99 = Extremely High]). See, for example, Ackerman and Wolman (2007) for a review of the constructs and their reliability and validity.

##### 4.2.1.2. Personality and motivational traits

Specific personality trait measures were constructed from the International Personality Item Pool (IPIP) collection of items and scales (Goldberg, 2008). Scales included Extroversion, Openness to experience, nAch, Social Closeness, Conscientiousness, and Self-Discipline. In addition, a scale of Boredom Proneness was included (for a review, see Vodanovich, 2003). For each of these scales, the response scale had six items (1 = Very Untrue of Me to 6 = Very True of Me).

##### 4.2.1.3. Typical Intellectual Engagement (TIE)

A 12-item short version of the Goff and Ackerman (1992) TIE scale was administered, with a response scale of 1 = Strongly Disagree to 6 = Strongly Agree.

##### 4.2.1.4. Motivational traits

The short form of the Motivational Trait Questionnaire (MTQ; Kanfer & Ackerman, 2000; see also Heggestad & Kanfer, 2000; Kanfer & Heggestad, 1997) is a 48-item measure that contains six scales. The scales represent markers for three underlying motivational trait factors: (1) approach-oriented motivation (Desire to Learn, Mastery), (2) competitive excellence (Other-referenced goals, Competitiveness), and (3) aversion-related motivational traits (Worry, Emotionality).

##### 4.2.1.5. Interest themes

The 90-item *Unisex Edition of the American College Testing Interest Inventory (UNIACT)* (Lamb & Prediger, 1981) was used to assess interest themes identified by Holland (1973) as Realistic (“accounting”), Investigative (“studying physics”), Artistic (“compose music”), Social (“run focus groups”), Enterprising (“entertain others”), and Conventional (“repair computers”). The response scale contained six items ranging from 1 = Strongly Dislike to 6 = Strongly Like.

In addition to the preceding measures, participants were asked to write (in response to open-ended questions) their top five leisure activities: “List your top five free-time/leisure activities (for example, socializing, playing video games, reading, exercising, volunteering, watching TV or movies, watching or playing sports, etc.)”.

#### Disutility judgments

A list of 125 activities that were designed to sample across both physically and mentally demanding tasks were created. Tasks were created using brainstorming by the authors, as well as suggested tasks from undergraduate staff members, from Holland’s Self-Directed Search “Leisure Activities Finder” (Holland, 1997b), and various Internet searches. The activities are listed in Table 1. The tasks were designed to broadly sample several different characteristics, such as different kinds of ability demands (verbal, spatial, numerical, perceptual speed) and different depths of demands, from low-stimulation tasks that would be expected to be rated as boring to high-demand tasks that would be expected to result in fatigue if performed over a period of time. “Four hours” was selected for the unit of time because it corresponds to the limit of work performed without a break in most laboratory study or employment contexts. In order to compare subjective mental effort to subjective physical effort performance, tasks were selected that varied in the degree to which they involved physical effort, in contrast to mental effort. Finally, tasks were selected to vary in terms of intrinsic interest. The physical activities ranged from relatively passive to highly active. Pilot testing was performed with a larger set of items, which were culled for participant familiarity, a wide range of disutility judgments, and to maintain adequate representation of each of the categories of tasks described above.

**Table 1:** Disutility judgments. Task items and descriptive statistics (mean, SD, 25%ile, median, 75%ile)

Item #	Item	Mean	SD	25^th^	Median	75^th^
				%ile		%ile
36.	Cleaning bathrooms	21.70	13.20	14.75	20.00	30.00
31.	Tutoring for SAT preparation	19.68	12.53	10.00	18.00	25.00
80.	Help people complete IRS tax forms	19.08	11.55	13.00	15.00	21.00
2.	Complete SAT-type essays	18.53	11.74	12.25	15.00	20.00
109.	Vacuuming and dusting or mopping floors	17.51	13.89	10.00	14.00	20.00
97.	Change flat tires on cars	17.18	12.61	10.00	15.00	20.00
108.	Waiting in an airport for a delayed flight	17.05	12.92	10.00	15.00	20.00
89.	Watching security cameras for a public park	16.89	10.99	10.00	15.00	20.00
12.	Evaluating different dorm/apartment rental insurance policies	16.87	10.70	12.00	15.00	20.00
65.	Determining the best allocation of investments for a retirement account (e.g., 401k)	16.53	14.13	10.00	15.00	20.00
46.	Helping people choose the best credit card to match their financial situation and expected expenses.	16.17	8.44	10.00	15.00	20.00
106.	Picking up trash along a park walking path	15.75	15.40	8.00	12.00	15.50
60.	Trim hedges/bushes	15.59	9.46	10.00	14.00	20.00
115.	Being a crossing guard at a school	15.29	10.37	10.00	15.00	20.00
58.	Entering data into Excel spreadsheets	15.26	11.01	9.50	12.00	20.00
113.	Canning vegetables	15.00	10.36	10.00	13.00	15.50
29.	Proofreading company documents for style, grammar, and spelling	14.97	8.99	10.00	15.00	20.00
17.	Washing cars	14.88	9.54	10.00	12.00	17.00
9.	Weeding a garden	14.80	10.02	10.00	15.00	20.00
32.	Filing patient charts/files in a doctor’s office	14.72	8.53	9.50	12.00	15.50
74.	Serving on a jury for a product liability lawsuit	14.41	12.96	9.50	15.00	18.00
8.	Painting an apartment living room	14.28	9.88	10.00	12.00	20.00
52.	Read textbook on European history	14.22	11.28	8.00	14.00	20.00
7.	Helping build a storage shed	14.20	8.48	10.00	13.00	20.00
114.	Presenting a request to a local planning commission	14.11	9.43	10.00	12.00	20.00
37.	Installing, setting up a new Wi-Fi router, and connecting various Internet-enabled devices (lights, thermostat, TV, etc.)	14.04	9.02	10.00	15.00	18.50
38.	Assisting in a bird census (observing, classifying, and counting birds in a nature sanctuary)	13.81	11.15	9.50	11.00	20.00
124.	Supervising children in a park	13.68	9.13	10.00	12.00	15.00
19.	Driving a car for 200 miles on an interstate highway	13.63	11.89	9.00	12.00	18.00
15.	Making a scrapbook for somebody you don’t know	13.54	7.70	10.00	13.00	16.00
26.	Serving on a jury for a trial where the defendant is accused of assault	13.36	12.78	6.75	10.00	19.25
51.	Making seating charts for a wedding	13.14	6.45	9.00	12.00	16.00
61.	Creating a monthly budget based on expenses over the past year	13.11	9.76	9.00	10.00	15.00
98.	Scanning print documents into the computer	13.03	9.32	8.50	10.00	15.00
121.	Leading a classroom discussion on assigned readings	12.80	7.09	8.00	11.00	19.00
43.	Re-finishing an old coffee table	12.69	7.86	9.00	10.00	15.00
5.	Obedience training a dog	12.63	10.65	8.00	10.00	15.00
99.	Write a short story	12.61	12.90	5.00	11.00	20.00
16.	Assisting in an archeological “dig”	12.57	13.97	7.25	14.00	20.00
44.	Designing and constructing a birdhouse	12.49	8.75	9.00	12.00	15.00
53.	Assembling layouts for a print newsletter (photos, text, advertising)	12.45	7.33	8.00	10.00	15.00
35.	Stuffing envelopes for a mass mailing	12.42	6.78	9.00	10.00	15.00
105.	Being a referee at an amateur tennis competition	12.33	9.86	8.00	10.00	15.00
27.	Rake leaves	12.18	7.58	8.00	10.00	15.00
70.	Writing poetry	12.15	12.87	5.00	12.00	19.00
93.	Listing items online for sale	12.15	8.27	8.00	10.00	15.00
96.	Organizing computer files for backup	12.11	8.03	8.00	10.00	15.00
95.	Planning the needed supplies and materials for a “poker night” for 100 people	11.98	8.08	8.00	10.00	15.00
50.	Do laundry	11.85	10.02	8.00	10.00	14.00
125.	Assembling IKEA-type furniture	11.85	11.63	8.00	10.50	15.00
3.	Addressing invitations for a wedding	11.80	5.35	8.00	10.00	15.00
49.	Entering phone/e-mail contact information into new smartphones	11.80	7.60	8.00	10.00	15.00
64.	Presenting a book report to a book club	11.79	10.04	6.50	10.00	15.00
116.	Using Photoshop to “restore” old photos	11.79	10.81	7.50	10.00	18.00
42.	Filing books in a public library	11.55	8.22	8.00	10.00	15.00
24.	Preparing/revising a résumé	11.49	8.24	8.00	10.00	15.00
112.	Organizing photos and text for a website	11.35	7.05	8.00	10.00	15.00
11.	Helping people move items in or out of a dorm room	11.31	8.25	8.00	10.00	15.00
45.	Researching cellphone plans and prices	11.17	6.51	8.00	10.00	14.50
73.	Making the schedule of games for an amateur sports tournament	11.07	6.41	8.00	10.00	15.00
118.	Sorting clothes for a rummage sale	11.00	8.12	7.00	10.00	15.00
33.	Researching prices and options for purchase of a computer printer	10.81	8.05	7.00	10.00	13.00
76.	Creating PowerPoint slides for a presentation	10.79	7.16	7.50	10.00	15.00
79.	Programming a robot for a 4th grade class demonstration	10.55	12.80	4.00	10.00	15.00
77.	Using online resources for researching event venues for a celebration	10.55	6.41	7.50	10.00	15.00
120.	Editing a video and adding audio tracks for a YouTube video	10.45	11.31	5.00	10.00	15.00
28.	Assembling a custom desktop computer according to printed and video instructions	10.22	13.15	3.25	10.00	15.75
86.	Writing thank you notes	10.13	7.73	5.00	10.00	15.00
54.	Hang posters and pictures on walls	9.98	6.65	7.00	10.00	12.00
85.	Grading 4th grade math problem sheets (e.g., addition/subtraction problems)	9.96	6.37	8.00	10.00	12.00
91.	Riding as a passenger for 200 miles on an interstate highway	9.92	12.24	3.25	9.00	14.75
23.	Planning a course for a 10K charity walk	9.88	7.75	7.00	10.00	15.00
84.	Sketching still life scenes	9.61	11.93	1.50	9.00	15.00
78.	Reading poetry	9.54	11.70	4.50	10.00	14.50
34.	Dog walking	9.48	6.44	7.00	9.50	14.50
41.	Planning a dinner party for 8 people (menu, grocery list, etc.)	9.44	7.98	5.50	10.00	12.00
123.	Planning a vacation (i.e., comparing prices and routes for airline travel and hotels)	9.37	10.20	5.00	10.00	15.00
1.	Completing a paint-by-numbers project	9.01	10.08	5.00	10.00	12.00
57.	Bird watching	8.84	10.03	.00	10.00	15.00
14.	Installing applications on a smartwatch	8.61	9.06	2.50	8.00	10.00
104.	Reading a biography about Franklin D. Roosevelt	8.40	10.76	.00	7.00	13.50
107.	Watch a documentary on the history of Afghanistan	8.32	10.58	.00	10.00	12.00
63.	Attending a lecture on art appreciation	7.93	11.22	.00	8.00	15.00
122.	Attending a political debate (e.g., for the governor’s office or for a senator)	7.70	14.25	.00	8.00	15.00
6.	Reading *Time/Newsweek/US News & World Report* magazines	7.64	8.62	.00	9.00	11.50
102.	Assembling a desk clock kit from printed instructions with small tools and a soldering iron	7.46	9.48	.00	9.00	12.00
**Minimum Wage Boundary**						
59.	Watching TED Talks on high energy physics	7.00	13.83	−.50	7.00	11.00
55.	Attending a lecture on film history	6.96	10.24	.00	8.00	12.00
40.	Watch a documentary on the fashion industry	6.79	9.44	.00	8.00	10.00
92.	Watch a documentary on detecting art fraud	6.45	11.50	−2.00	7.00	11.50
56.	Judging an audition for a talent show	6.29	10.60	.00	6.00	10.00
72.	Solving Rubik’s Cubes	6.15	10.54	.00	8.00	12.50
18.	Read *The New York Times*	6.00	8.21	.00	7.00	10.00
111.	Watch a documentary on the Flint, Michigan water crisis	5.85	10.17	.00	5.00	10.00
13.	Creating a playlist of songs for a party/event	5.62	8.25	.00	5.00	10.00
100.	Playing chess	5.29	11.16	−1.50	5.00	10.00
47.	Assembling a complex jigsaw picture puzzle	5.25	8.70	.00	5.00	10.00
69.	Completing crossword puzzles	5.07	7.06	.00	5.00	10.00
66.	Playing bridge	4.96	7.97	.00	5.00	10.00
30.	Completing Sudoku puzzles	4.74	8.69	.00	5.00	10.00
22.	Watch a documentary on the Large Hadron Collider	4.58	7.31	.00	5.00	10.00
110.	Read Stephen Hawking’s *A Brief History of Time*	4.49	9.95	−.50	5.00	10.00
20.	Playing Solitaire on the computer	4.41	8.91	−1.00	5.00	8.00
10.	Playing Scrabble	4.07	8.55	−1.00	5.00	8.00
4.	Play Bingo	3.77	7.25	−1.50	5.00	9.50
48.	Searching for ancestry information (e.g., looking up your distant relatives on Ancestry.com)	3.65	10.45	−5.00	2.50	10.00
75.	Attending the opera	3.50	16.52	− 5.50	5.00	13.00
87.	Attending a workshop on financial literacy (for example, credit cards, mortgages, life insurance, retirement)	3.22	14.45	−6.00	.00	10.00
81.	Playing Trivial Pursuit	2.93	7.71	.00	1.00	8.00
88.	Learning how to juggle	2.79	10.45	−4.50	.00	8.00
68.	Playing a Tetris-type game	2.76	6.57	−1.00	.00	8.00
103.	Attending a health and wellness workshop for planning a personal exercise program	2.59	10.54	− 4.50	.00	10.00
39.	Playing Jeopardy! with other college students	2.50	9.09	−4.00	.00	9.50
94.	Learning how to control a flying drone	2.37	10.98	−5.50	2.00	3.00
119.	Playing strategy games such as Risk or Settlers of Catan	2.28	8.41	−2.00	.00	8.00
101.	Read mystery novel	2.27	10.17	− 2.50	.00	8.00
71.	Watching TED Talks on bioengineering (e.g., new portable diagnostic devices, tissue engineered organs)	2.03	9.57	−4.00	.00	10.00
67.	Attending a lecture on artificial intelligence	1.96	10.43	−9.00	.00	5.00
117.	Watching TED Talks on motivation and learning	1.96	8.32	−3.00	.00	10.00
21.	Watching TED Talks on human relationships	1.80	7.18	− 3.00	.00	8.00
83.	Visiting an art museum	.26	10.65	−7.50	.00	8.00
62.	Attending a play by Shakespeare	.24	11.93	−8.50	.00	9.00
90.	Attending a classical music concert	.20	15.33	−10.00	.00	10.00
82.	Playing pool (billiards)	.16	6.90	−5.00	.00	5.00
25.	Watching TED Talks on new technology advances	.06	7.46	−5.00	.00	6.50

Participants were presented with each activity separately and asked to provide the “minimum amount of money per hour” they would require to be willing to perform the task over a 4-h period. Participants were given examples and instructed that if they would do the task for free, they should enter zero dollars per hour, and if they would pay to do the task, they should enter a negative amount. For the purpose of anchoring their responses, participants were informed that the “Federal minimum wage is $7.25/hour. So, if you consider the task listed to be like ‘work’, then you should expect at least minimum wage to do the task.” The assumption that the rate of pay reflects perceived effort is reasonable, given that empirical findings have implied that the value of task engagement can be derived from decisions based on monetary compensation (e.g., COGED paradigm; Westbrook et al., 2013) or cost-benefit analysis (Kurzban, Duckworth, Kable, & Myers, 2013).

#### Similarity judgments

Multidimensional scaling (MDS) is a statistical technique used to provide a spatial representation of participants’ perceptual space (for an introduction to the technique, see Kruskal & Wish, 1978; see also Dunn-Rankin, Knezek, Wallace, & Zhang, 2004). The technique is most useful when one has a set of stimuli that may differ on multiple dimensions, but only some stimulus dimensions may be relevant for judgments made by people, when considering issues such as attractiveness, likability, disutility, or many other evaluative considerations. The MDS technique has received use in a variety of different domains, including basic research on perception and psychophysics, but also in marketing (in terms of determining salient product characteristics), social psychology (in terms of social networks), and others. Participants are instructed to view pairs of stimuli, and to provide a similarity or dissimilarity judgment of each pair, either in an undefined manner or with respect to a specific construct. The major difficulty associated with this technique is that it generally requires pairwise comparisons of all of the stimuli in a set, so that as the number of stimuli increases, the number of unique judgments that are required increases exponentially (where *n* is the number of stimuli, the total number of unique pairs for comparison is (*n* * *n* – 1)/2. Thus, a set of paired comparisons for the entire set of 125 tasks would require 7750 judgments, far beyond the patience of participants.

In order to make the similarity judgment process manageable in terms of time and effort on the part of the participants, a subset of 24 items from the original list of 125 activities was selected for this part of the study. The items included a representative sample of the activities, designed to maximize the coverage of different types of mentally and physically demanding tasks. The items selected for this part of the study are listed in Table 2. Participants were presented with each item paired with every other item, in a Ross (1938) order, which maximizes the number of different intervening items between judgments. The participants were instructed to “Rate how SIMILAR the tasks are, with respect to how much effort they would require from you, in order to perform them. If both tasks would require about the same amount of effort from you, you should rate them as ‘similar.’ If they require different amounts of effort from you, rate them as ‘different’.” The response scale used for these judgments ranged from 1 (Extremely Different) to 8 (Extremely Similar), with explicit adjectives that were perceptually approximately equally spaced (Cliff, 1959) for each of the whole-number response options. A total of 276 ((24 × 23)/2) stimulus pairs were administered for the similarity judgments. Also, to assess the reliability of similarity judgments, the first 25 pairs of stimuli were repeated at the end of the similarity judgment section.

**Table 2:** Multidimensional scaling (3D) of similarity judgment items (ordered by attractiveness/aversiveness, from preference judgments)

				MDS solution		
Abbreviation	Item		Attractiveness/aversiveness	I	II	III
pool	82	Playing pool (billiards)	20.05	.070	−1.116	−.056
tetris	68	Playing a Tetris-type game	22.77	.165	−.977	−.116
jigsaw	47	Assembling a complex jigsaw picture puzzle	24.56	.551	−.512	−.329
sudoku	30	Completing Sudoku puzzles	27.40	.571	−.561	−.068
chess	100	Playing chess	28.55	.760	−.679	−.038
vacplan	123	Planning a vacation (i.e., comparing prices and routes for airline travel and hotels)	33.22	.515	.801	−.381
prog4th	79	Programming a robot for a 4th grade class demonstration	36.36	1.107	−.192	−.401
readNYT	18	Read *The New York Times*	37.27	−.167	−.434	.790
birdhous	44	Designing and constructing a birdhouse	39.78	.731	.077	−.846
**Boundary between Attractiveness/Aversiveness**						
orgbkup	96	Organizing computer files for backup	45.03	−.035	.455	.018
rpoetry	78	Reading poetry	47.38	.182	−.638	.583
movedorm	11	Helping people move items in or out of a dorm room	50.82	−.445	.468	−.946
thankyou	86	Writing thank you notes	51.86	−.682	.116	.624
excel	58	Entering data into Excel spreadsheets	52.32	−.479	.426	.360
laundry	50	Do laundry	53.82	−1.045	−.108	−.255
filing	32	Filing patient charts/files in a doctor’s office	55.70	−.631	.294	.153
401k	65	Determining the best allocation of investments for a retirement account (e.g., 401k)	56.48	.661	.919	.188
stufenv	35	Stuffing envelopes for a mass mailing	60.25	−.976	.018	.132
trash	106	Picking up trash along a park walking path	61.22	−.948	−.093	−.613
seccam	89	Watching security cameras for a public park	62.73	−.922	−.236	.472
weeding	9	Weeding a garden	63.42	−.587	.152	−.891
readEH	52	Read textbook on European history	64.45	.389	.249	.863
irs	80	Help people complete IRS tax forms	69.95	.257	.973	.228
satessay	2	Complete SAT-type essays	77.39	.958	.596	.526

Notes: *Dimension I* Tedious vs. Challenging, *Dimension II* Games vs. Work/Education/Chores, *Dimension III* Physical vs. Mental Demands

#### Preference judgments

Preference judgments were solicited for each of the 24 tasks administered in the similarity judgment section. Participants were instructed to “Rate each task in terms of how much you would like or dislike performing the task for a 4-hour period.” The response scale ranged from 0 (Very Attractive) to 100 (Extremely Aversive), with the dividing line between attractive and aversive equal to 35. That is, the response scale was not symmetric, in that none of the tasks were expected to be extremely attractive and some of the tasks were expected to be extremely aversive.

### Procedure

The at-home questionnaire was handed out to participants at least 24 h prior to the laboratory session. For the laboratory session, participants completed the judgment tasks on 19-in. laptop computers, where the stimuli were presented using Qualtrics software in a self-paced format. The participants first completed the disutility judgment section, followed by the similarity judgment section and the preference judgments. A 5-min break was provided halfway through the similarity judgment section. After the preference judgments were completed, participants were debriefed and excused from the study.

## Results

The results of the study represent several different aspects of the perceptions related to the disutility of mentally and physically demanding tasks. In the first section, the basic descriptive statistics of the disutility estimates for the 125 activities provide a window into the perceived attractedness or aversion to the tasks. Game-like tasks such as playing Tetris, for example, have a distinct set of characteristics compared to chore-like items such as filling out IRS forms. In the second section, MDS analysis of the similarity judgments of the 24-activity subset was performed to indicate the underlying subjective perceptual space for the similarity and differences among these tasks, and the MDS solution was used as the basis for indicating the location of preferences for the preference judgments. In the third section, modeling of preference judgments was used to determine both the location of preferences for individual participants and the underlying nature of their preference space (e.g., vector or ideal point). Finally, in the fourth section of the results, the three types of judgment data (disutility, similarity, and preferences) were examined with respect to individual differences in traits, task specific self-efficacy, and self-concept variables. Each of these is treated in turn in the following sections.

### Disutility judgments

Before analysis of the disutility judgments, the data were “cleaned” by setting as missing any disutility estimate of $100/h or higher. These values were deemed either not serious estimates by the participants or highly unrealistic (given that they correspond to an annual salary of $208,000). None of the activities we asked to be rated would correspond to such a salary (e.g., such activities would be hedge fund trading and cardiac surgery). Of the 6625 ratings provided by the participants, only 25 (or 0.3%) of the ratings were eliminated by this criterion.

Descriptive statistics for the disutility judgments are shown in Table 1, including mean, standard deviation (SD), and 25th percentile, median (50th percentile), and 75th percentile values, in dollars/hour. There are several interesting aspects of these results, given that no similar estimates are found in the literature.

First, the disutility judgments are essentially normally distributed, given that the means and medians are highly similar. Second, there is a wide range of disutility estimates across the range of activities that were administered—from the highest disutility (cleaning bathrooms) to the lowest disutility (watching TED Talks on new technology advances). Third, 39 of the 125 activities were rated as having a lower disutility than would be associated with minimum wage (i.e., less than $7.25/h). Fourth, participants rated many activities as having a zero disutility or a positive utility (meaning that they would do the activity for free, or pay to do the activity). While none of the 125 activities had an average positive utility, all of the activities with a mean disutility below minimum wage had at least 25% of the participants willing to do the activities for free or willing to pay to do the activity. These activities represented an interesting cross section of tasks, but they mainly pertained to reading, game playing, watching videos (TED Talks, documentaries), and attending cultural activities (art museum, opera).

In contrast, the activities with high disutility values included physically demanding tasks (e.g., trim hedges/bushes, change flat tires on cars), tedious perceptual speed tasks (e.g., filing patient charts, watching security cameras for a public park, proofreading), and highly mentally demanding tasks (e.g., complete SAT-type essays, determine the best allocations of investments for a retirement account).

#### Subjective effort-disutility function (Hypothesis 1)

Prior to the study, seven staff members reviewed and rated 100 of the mentally demanding tasks (i.e., this did not include the physically demanding tasks) regarding the effort demands of the tasks. The rating form ranged from 1 to 10; at the low end were tasks described as “boring... very little stimulation... Typically the individual would be passive (attending, rather than responding)”, in the middle were tasks that were neither boring nor fatiguing, and at the high end were tasks “likely to lead to feeling of high mental effort expenditures and after time, mental fatigue.” Average inter-rater reliability for the subjective effort ratings was not high (range .27–.59), but the use of seven independent raters resulted in robust mean ratings (α = .84).

**Fig. 1:**
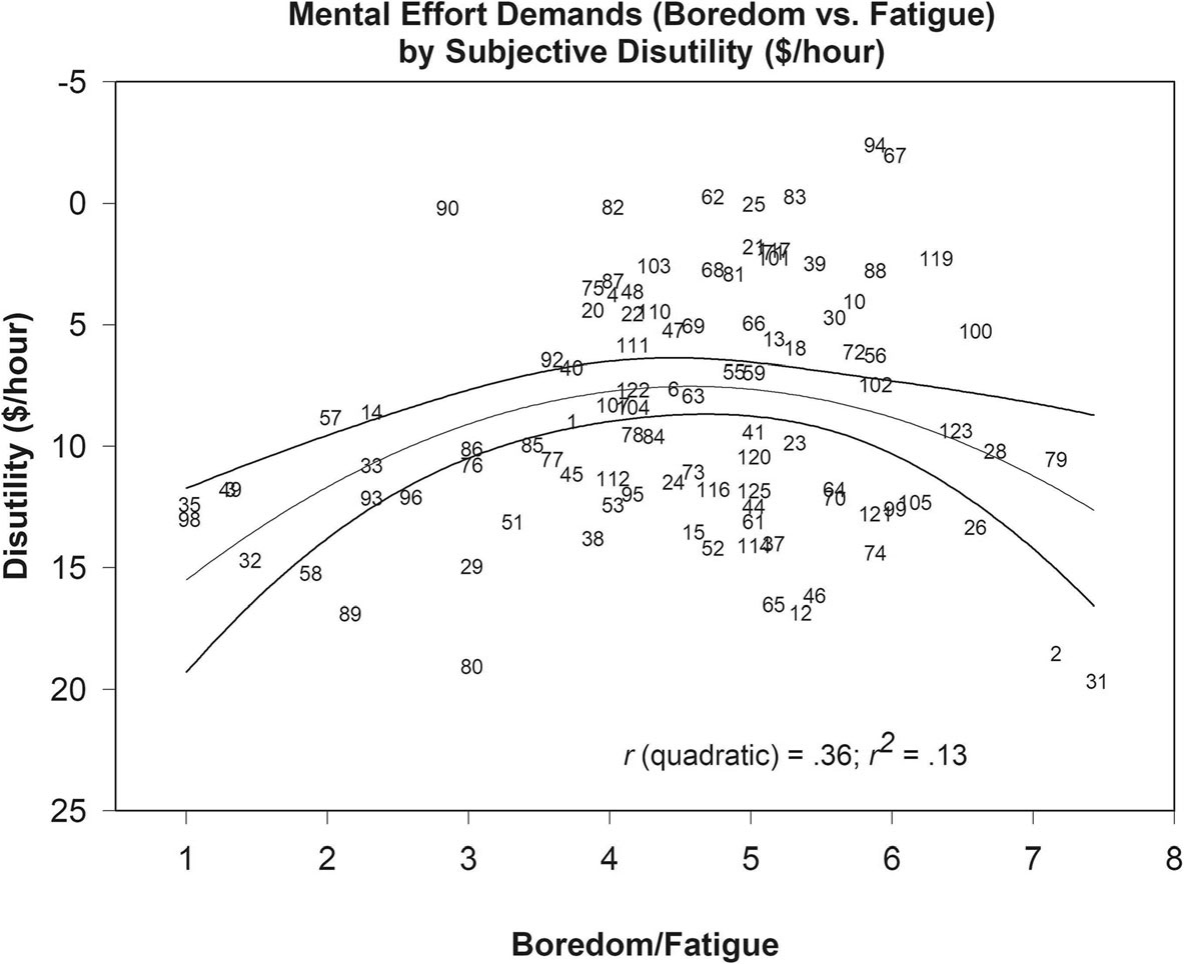
Rated task mental effort demands (boredom vs. fatigue) plotted against mean subjective disutility ($/hour) values. Quadratic regression shown with 95% confidence intervals as *solid lines*. *Numbers* indicate the task designation in Table 1. Disutility ratings *N* = 77, Boredom vs. Fatigue *N* = 7

Average ratings were plotted against the participant mean disutility ratings from the participants. The plot of subjective effort-utility for these tasks is shown in Fig. 1, where negative values on the ordinate indicate high subjective utility (i.e., participants would pay to be able to perform the task). Although there is a spread of disutility estimates across the various levels of boredom/fatigue, a quadratic regression (*r* = .36, *p* < .01) provided a better fit to the data than a linear regression (*r* = .16, *p* > .05). The test for incremental fit from a linear to a quadratic function yielded a significant result (*F* (1,97) = 11.71, *p* < .01), indicating that there is an inverted-U-shaped function relating effort to mean subjective disutility for this set of 100 tasks/activities.

Additionally, the data were subjected to Akaike information criterion (AIC) and Bayesian information criterion (BIC) analyses. These analyses correct for model complexity to show that the choice to use a higher order polynomial model is justifiable in terms of parsimony (Burnham & Anderson, 2004). The BIC score for the quadratic model was lower than that for the cubic model (*BIC*_quadratic_ = 610.29, *BIC*_cubic_ = 610.38), indicating that a quadratic model is the most parsimonious fit for the data. However, the AIC indicated that a cubic model is the most parsimonious fit for the data, given that the AIC score for the cubic model was lower than it was for the quadratic model (*AIC*_quadratic_ = 599.87, *AIC*_cubic_ = 597.36).

We resolved the conflict between selection criteria by choosing a quadratic model for two reasons. First, the BIC penalizes complexity more heavily and is therefore the more “conservative” selection criterion (Chou & Reichl, 1999). Second, the observed BIC and AIC scores are in disagreement, with only slight differences between the quadratic and cubic models in each case. When these statistics recommend competing models, it may suggest a “range of acceptable models” (Kuha, 2004, p. 222). In this case, both statistics clearly suggest that polynomial models are a better fit than a linear model (*BIC*_linear_ *=* 617.08, *AIC*_linear_ = 609.27). Given the choice between two acceptable models, the quadratic model allows for a more parsimonious interpretation of the Effort-Utility curve. Together, the model comparison analyses provide support for Hypothesis 1, indicating that the Effort-Utility function is characterized by an inverted-U shape, with the most attractive tasks requiring a moderate amount of effort.

#### Similarity judgments: MDS (Hypothesis 2)

**Fig. 2:**
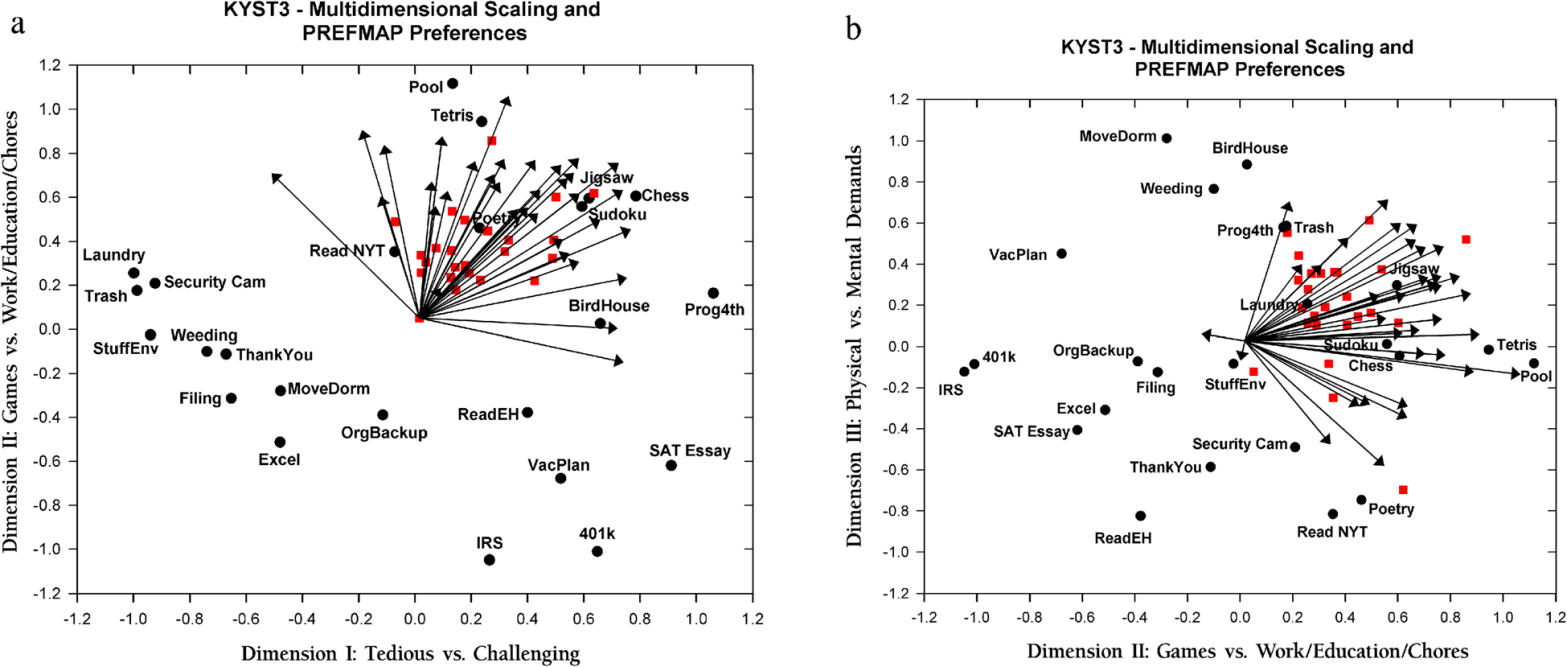
Multidimensional scaling solution and preferences (vectors and ideal points). **a** Dimension I (Tedious vs. Challenging) vs. Dimension II (Games vs. Work/Education/Chores). **b** Dimension II (Games vs. Work/Education/Chores) vs. Dimension III (Physical vs. Mental Demands). Tasks are shown as *black circles*. Preference vectors are shown as *arrows*. Ideal points are shown as *red squares*. *N* = 77

To obtain a general solution to the question of the perceptual space for the 24 activities, mean proximities were computed for each pairwise judgment, and the proximity matrix was then subjected to KYST-3 non-metric MDS (Kruskal, Young, & Seery, 1973).^2^ Solutions ranging from four dimensions to one dimension were derived. Stress Formula 1, which indicates the degree of fit to the data (larger numbers indicate a poorer fit) indicated that a three-dimensional (3D) solution represented the best combination of fit and parsimony to the data (4 dimensions, Stress = .081, 3 dimensions, Stress = .114, 2 dimensions, Stress = .194, 1 dimension Stress = .382). The 3D solution was rotated to a principal components orientation, and is provided in Table 2 and Fig. 2a and b.

From the solution shown in the table and figures, tasks that are perceived as very similar in effort requirements by the participants are close in proximity to one another (e.g., “playing Sudoku” and “playing chess” or “weeding a garden” and “helping people move items in or out of a dorm room”). In contrast, those tasks that are perceived as very different in terms of effort requirements (e.g., “playing pool” vs. “complete SAT-type essays”) are far away from one another in the MDS solution.Identification of the dimensions from an MDS solution is often a subjective process, but in this case we also referred to ratings data from staff members that pertained to ratings of tediousness/fatigue, physical/mental demands, knowledge demands, level of intrinsic interest, and mental content (verbal, spatial, numerical, perceptual speed). Based on this information and a review of the MDS solution, the dimensions were labeled as follows: Dimension I: Tedious vs. Challenging Tasks, Dimension II: Games vs. Work/Education/Chores, and Dimension III: Physical vs. Mental Demands. In other words, the normative description of perceptual space for these participants is that they perceive the similarity and differences in effort required to perform the tasks along the lines of these three dimensions. This supports Hypothesis 2, which predicted the multidimensional nature of task perception.

### Preference judgments

#### Hypothesis 3

In order to determine individual preferences for task types (i.e., to provide a perspective on the Effort-Utility function), the preference judgments can be resolved with reference to the common MDS solution. PREFMAP (Meulman, Heiser, & Carroll, 1986) is a program that estimates preferences using a hierarchy of models. The first model tested for each participant is the vector model. A vector model corresponds to a “more is better” set of preferences. That is, depending on the direction of the vector in the stimulus (MDS) space, an individual’s preference would be for more or less of the underlying constructs identified in the space. For example, an individual who preferred only activities that were more game-like (Dimension II), and who also didn’t care about whether the tasks had physical or mental demands (Dimension III) or whether the tasks were tedious or challenging (Dimension I), would have a vector that had high negative values for Dimension II and zero values for Dimension I and Dimension III. If the individual was well fitted by the vector model, then that person’s most-preferred task would be one outside of the current set of task stimuli.

The next model in the hierarchy models is the “ideal point” model. A participant whose preferences are well fitted by this model has the highest preference for a particular kind of task within the stimulus space, represented as a single point in the space. Tasks that are closest to the ideal point will be most preferred, and tasks that are furthest away will be least preferred. If the individual’s ideal point is close to one or more tasks, then those tasks represent the individual’s highest preference.

Two additional models are tested, both of which are variations of the ideal point model.

The “weighted unfolding” model allows for individuals to have differential weights for the underlying MDS dimensions in determining the gradient of preferences. That is, two individuals might have the same or similar ideal points but differential weights for the underlying dimensions. A higher weight on a dimension means that a steeper decline in preference would be expected as the task is more distant on that dimension, but a comparatively shallower decline in preference would be expected for tasks that only differ on dimensions with smaller weights.

The last model, called “general unfolding”, allows not only for weights to differ for the dimensions, but also allows for the stimulus space to be rotated to different orientations for each individual. The PREFMAP program allows for testing of all four preference models and for incremental testing of the model fit for each model in the hierarchy.

The results of the PREFMAP modeling indicated that 17 participants were not well fitted by any of the models, 35 were best fitted with a vector model, and the remaining 25 were best fitted by one of the ideal point models. Illustrations of the locations of the vectors and ideal points for the participants who were well fitted are shown in Fig. 2a and b. To determine an individual’s preferences among the various tasks, one need only evaluate the projections of the stimuli onto the individual’s preference vectors (for participants best fitted by a vector model) or calculate the distance between the individual’s ideal point and the stimuli, across the three MDS dimensions (for those participants best fitted by an ideal point model).

Although there was substantial variability in both the vectors and ideal points across participants, most of the participants had preferences that had moderate positive values on Dimension I (Tedious vs. Challenge) and negative values on Dimension II (Games vs. Work/Education/Chores). For Dimension III (Physical vs. Mental), there was more variability across participants. Ten of the participants had vectors or ideal points that were loaded on the Mental side of the Physical vs. Mental dimension, while the other 51 participants were loaded on the Physical side of the dimension. None of the participants was oriented toward more intense physical tasks (e.g., “weeding a garden” or “helping move into/out of a dorm”), but rather they preferred tasks more similar to “playing pool” or “putting together a complex jigsaw puzzle”). Together, these results support Hypothesis 3, which predicted that participants would differ as to whether their preferences are best characterized by a vector model or an ideal point model.

#### Preference judgments and disutility judgments

Although deriving subjective preference space through the initial MDS and subsequent preference model analysis provides both a multidimensional and a vector/ideal point description of the participants’ judgments, it is useful to also look at the congruence between the direct estimates of preferences and the disutility judgments that participants made in the first component of the study. For this purpose, the mean disutility estimates (in terms of dollars/hour required for the participant to do the task for 4 h) were regressed against mean preference judgments for the 24 tasks that were common to both study components.

**Fig. 3:**
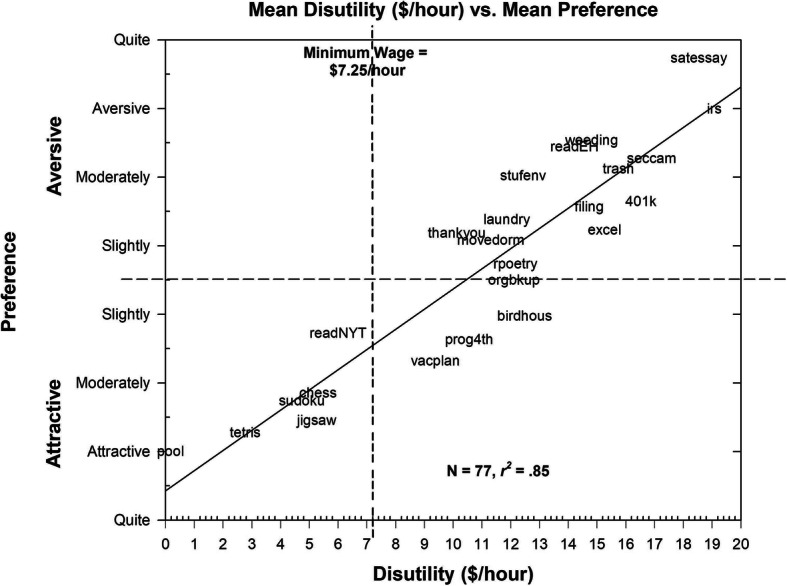
Mean disutility ($/hour) plotted against mean preference values. *Dashed lines* indicate boundaries for minimum wage (on the abscissa) and between ratings of attractiveness and aversiveness (on the ordinate). *N* = 77. Linear regression shown with *solid line*. Tasks are shown as abbreviated labels from Table 2

The result is shown in Fig. 3. The degree of association between these two sets of variables is substantial and linear, with the correlation between the two sets indicated as *r* = .92 (*p* < .01). In addition, by triangulating the disutility judgments and the attractive/aversive categories of the preference judgments, one can examine the similarities and differences between the different kinds of estimates. Even though none of the mean disutility judgments were negative (which would have indicated that participants would be, on average, willing to pay to do the activity in question), nine of the activities were, on average, rated to be Slightly Attractive to Attractive. Six of these activities—five games and “reading *The New York Times”*—were rated with disutility values below the federal minimum wage. Although the minimum wage may be a somewhat arbitrary value, the fact that, on average, participants were willing to engage in these activities for less than minimum wage compensation suggests a rough equivalence between the mean preference judgment of “attractiveness” and the mean disutility judgments. In contrast, all of the tasks rated as Slightly Aversive to Quite Aversive corresponded to disutility estimates substantially above minimum wage.

When compared with the MDS solution loadings for the 24 activities common to the disutility judgments and dissimilarity judgments, only the correlation with Dimension II (Games vs. Work/Education/Chores) was significant (Dimension I: *r* = .21, *ns*; Dimension II: *r* = .77, *p* < .01; Dimension III: *r* = .12, *ns*). That is, even though the participants discriminated the effort required by these activities along three different dimensions, the only dimension of perceptual space significantly relevant to judgments about disutility was whether the activities were games or associated with work, education, or chores.

### Trait and other correlates of task preferences

#### Trait indicators

The at-home questionnaire contained multiple measures of several broad non-ability constructs that we hypothesized might be related to subjective disutility and preference judgments, namely self-concept, vocational interests, and personality/motivation. Trait measures, along with item numbers, means, SDs, and internal consistency reliability estimates are provided in Table 3. Calculating correlations between each of these scales and individual task preferences would pose a substantial problem of Type 1 statistical errors, given the sheer number of scales and activities. In order to bring the number of comparisons under control, to capitalize on the communality among both trait measures and preference measures, and to take advantage of the principle of aggregation (e.g., see Rushton, Brainerd, & Pressley, 1983) for non-ability measures, we consolidated the trait measures, based mainly on factor analysis and analysis of respective internal consistency reliability estimates (e.g., see Ackerman, 2003 for a discussion of this approach to non-ability predictions of performance and attitude criteria). The scales and composites are described below and in Table 3.

**Table 3:** Trait measures: number of items, means, reliabilities, and internal consistency reliability

Variable	Items	Mean	SD	α
Self-Concept and Self-Estimates of Abilities				
Verbal Self-Concept	6	5.00	.65	.76
Math Self-Concept	6	5.15	.73	.86
Spatial Self-Concept	6	4.72	.70	.79
Self-Estimate of Verbal Abilities	4	71.59	14.03	.79
Self-Estimate of Math Abilities	5	77.36	13.87	.88
Self-Estimate of Spatial Abilities	4	68.04	15.85	.84
Self-Estimate of Perceptual Speed/Psychomotor Abilities	6	69.13	15.11	.87
Self-Estimate of General Abilities	3	71.10	14.09	.75
Preference for Numerical Information	11	4.68	.75	.88
Composites				
Verbal/Crystallized	5	0.00	1.00	.83
Math/Fluid	4	0.00	1.00	.75
Personality and Motivation				
Typical Intellectual Engagement	12	4.17	.76	.85
Extroversion	10	3.90	.93	.90
Openness to experience	12	4.08	.68	.72
Need for Achievement	10	4.68	.63	.84
Social Closeness	10	4.17	.98	.90
Conscientiousness	12	4.25	.73	.87
Self-Discipline	10	3.05	.84	.90
Boredom Proneness	7	2.87	.66	.65
Desire to Learn	8	4.61	.67	.83
Mastery	8	4.21	.47	.83
Other-Oriented Goals	7	4.16	.76	.79
Competitiveness	6	3.40	.37	.90
Worry in Achievement Contexts	10	3.75	.69	.86
Emotionality in Achievement Contexts	9	3.45	.62	.86
Composites				
Mastery/nAch	5	0.00	1.00	.89
TIE/Openness, Desire to Learn	3	0.00	1.00	.77
Extroversion, lack of worry in Achievement context	4	0.00	1.00	.70
Competitiveness, Other-oriented goals	2	0.00	1.00	.72
Interests				
Realistic	15	51.45	13.46	.89
Investigative	15	56.21	13.82	.91
Artistic interests	15	54.36	16.27	.90
Social	15	61.79	10.43	.82
Enterprising	15	52.08	12.46	.87
Conventional	15	47.23	15.11	.92

##### 5.3.1.1. Composites

The nine self-concept and self-estimate of ability scales were combined into two composites. The result was two composites for self-concept/self-estimates of ability (a Verbal/Crystallized self-concept composite and a Math/Fluid ability self-concept composite), and the Personality and Motivation traits were combined in four composites, as follows: (1) Mastery/need for Achievement, (2) Typical Intellectual Engagement (TIE), Openness, Desire to Learn, (3) Lack of worry/emotionality in achievement contexts, Social Closeness and Extroversion, and (4) Competitiveness and Other-oriented goals). In addition, the six vocational interest scales were considered as separate constructs, for a total of 12 trait measures. As can be seen in Table 3, the internal consistency reliability/homogeneity indicators (Cronbach’s α) for these various composites were generally slightly lower than the narrower individual scales, as expected, but they do indicate that the composite measures are coherent and robust indicators of broader traits.

##### 5.3.1.2. Preference composites

The 24 task preference measures were also aggregated for the purpose of this analysis, based on a similar factor analysis and examination of internal consistency reliability estimates. Eight tasks/activities did not easily fit into the groups and were thus eliminated from this analysis. Four coherent sets of activities were identified as: (1) Perceptual Speed/Physical (6 tasks, α = .78), (2) Games (4 tasks, α = .69), (3) Retirement Account Planning and IRS form assistance (2 tasks, α = .81), and (4) Reading/Writing (e.g., European history, New York Times, Writing SAT essays) (4 tasks, α = .59).

#### Hypothesis 4: cross-correlations between traits and preferences

Correlations were computed between the 12 trait/composite measures and the mean preference scores for the preference group data. The results are shown in Table 4. Note that because tasks/activities judged as less attractive/more aversive have larger numbers, positive correlations between traits and task preferences indicate that participants with higher trait values reported less attraction/more aversion to the tasks/activities.

**Table 4:** Trait correlates of task/activity preferences

Trait	Task/Activity			
	Group 1	Group 2	Group 3	Group 4
	PS/physical	Games	Retire plan	Read EH,
			IRS	NYT, write
				SAT essays
Self-Concept				
Verbal/Crystallized	.02	.10	−.06	−.24*
Math/Fluid	.25*	−.25*	−.22	−.00
Interests				
Realistic	−.15	−.20	.01	−.10
Investigative	−.05	−.01	.14	−.39**
Artistic	−.11	−.12	.44**	−.37**
Social	−.15	.12	.07	−.16
Enterprising	.29*	.25*	−.43**	−.34**
Conventional	.05	−.12	−.57**	−.12
Personality Trait Complexes				
Mastery/Need for Achievement	.06	.00	.03	−.29*
TIE, Openness, Desire to Learn	.16	−.17	.06	−.49**
Lack of worry/negative emotions in Ach Contexts, Social Closeness, Extroversion	.31**	.26*	−.24*	−.22
Competitiveness, Other-Oriented Goals	.16	−.21	−.09	.12

Positive correlations indicate higher levels of trait are associated with lower attractiveness/higher aversion to tasks/activities, negative correlations indicate higher levels of trait associated with higher attractiveness/lower aversion to tasks/activities

*PS* Perceptual Speed, *IRS* Internal Revenue Service, *EH* European history, *NYT The New York Times, Ach* Achievement

*p < .05; **p < .01, *N* = 77

Although many of the correlations did not reach statistical or meaningful levels of magnitude, some of the correlations were substantial in magnitude, and they present a coherent view of the correspondence between traits and task attractiveness/aversiveness. For self-concept, Verbal/Crystallized abilities were associated with more positive preferences for the Reading/Writing group of tasks, while Math/Fluid abilities were associated with greater aversion to Perceptual Speed/Physically Demanding tasks/activities and less aversion to Games and Retirement account planning and IRS form assistance. For personality traits, participants who were high on Mastery/Need for Achievement and TIE/Openness/Desire to Learn had less aversive ratings of the Reading/Writing tasks/activities, while those high on Lack of Worry/Emotionality in Achievement Contexts had higher aversiveness ratings for Perceptual Speed/Physical tasks and Games and slightly lower aversiveness for the Retirement account planning and IRS form assistance tasks.

The most substantial correlations (e.g., *r* = .3–.6) were found for the vocational interest measures, with participants high in Artistic interests having more aversion to the Retirement/IRS items and lower aversion to Reading/Writing items; those high on Investigative interests also had lower aversion to Reading/Writing items, while those with high levels of Enterprising and Conventional interests had lower aversion to the Retirement/IRS items. Together, these findings support Hypothesis 4, which predicted significant correlations between trait measures and task preferences.

#### Free-time activities

Participants were asked to list their “top five free-time/leisure activities.” Responses were coded by categories. The frequencies of the most popular activities were examined to provide some illumination between their preferences and utilities and what activities they actually engaged in when they had no other obvious constraints. From the 77 participants, the most highly endorsed categories were as follows: Watching Videos (*N* = 65), Exercise and Sport (*N* = 57), Socializing/Hanging with Friends or Family (*N* = 55). In contrast, Reading/Writing was only endorsed by fewer than half of the participants (*N* = 30).

## Discussion/conclusions

One of the primary goals of this study was to evaluate whether the subjective Effort-Utility function can be accurately described as having an inverted-U shape. From the current data, the answer is “yes.” Across the 100 mentally demanding tasks, the mean disutility estimates yielded a quadratic curve that clearly supports the notion that the highest utilities/lowest disutilities are associated with tasks of moderate levels of mental effort demands. On closer inspection, additional facets of these data are notable. First, there is substantial variance in mean utilities for tasks of similar effort demands, such that some moderately demanding tasks are viewed as much higher or lower disutility than the mean. Second, although the study is limited to a sample of college students, the utilities associated with particular tasks provide potentially revealing information. Mean utilities for nearly all of the tasks indicated that the average participant would not engage in the task for no compensation, even though many individuals would do so, including playing various games (e.g., Scrabble, chess, Sudoku) and watching educational videos or attending lectures on a variety of topics. However, it should be noted that this is a sample of university students at a selective institution, and as such, may not be highly representative of a random sample of young adults, or those in countries with substantially different work compensation conditions. Nonetheless, this study describes both the preferences among a group of undergraduate students (the most common population for psychological studies in the USA) as well as a methodology that can be adapted for other samples.

Cleaning bathrooms is understandably a task that has low desirability, but the fact that it is more aversive than completing SAT-type essays or waiting in an airport for a delayed flight provides some potential insight into the relative aversiveness of such tasks. Driving a car for 200 miles on an interstate highway was judged as much more aversive than riding as a passenger on the same trip, and reading news magazines was also seen as somewhat aversive. Such aspects of subjective utilities might represent characteristics of young adults that differ from those of older generations, something that might be explored in future investigations about aging and cohort differences. Other tasks judged as substantially aversive by most participants included determining the best allocation of investments for a retirement account (401k)—a finding that may have significant implications for financial literacy and well-being.

The MDS solution supports the proposition that subjective perceptions of effort are not just a unidimensional judgment of the total amount of effort needed to complete a task, but that they are a complex function of determinations of whether the tasks are likely to be tedious or challenging, whether the kind of effort demanded is primarily mental or physical, and whether the task is game-like or viewed as work, education, or a chore. When preferences are overlaid on the MDS solution, it is clear that most participants, whether vector-fitted or ideal point-fitted, are oriented toward mentally demanding game tasks, in contrast to tasks that are low effort or high effort, but that represent chores, work, or educational tasks.

It is worth noting that, given the variation found in terms of preferences characterized by a vector model vs. an ideal point model, individuals likely differ with respect to the dimensionality of their task preferences. Certain individuals are likely to enjoy engaging in tasks oriented toward a particular dimension (e.g., games) regardless of how the task is characterized by other dimensions. Other individuals are more likely to be engaged by a particular task if it falls at a somewhat optimal point on the dimension’s spectrum. For example, a person might be drawn to Tetris because it is moderately challenging (i.e., neither too tedious nor too difficult). Additionally, some individuals might prefer tasks that combine multiple dimensions. Therefore, these different preferential tendencies should be considered by researchers who desire to predict the extent to which individuals will engage in particular tasks or careers that individuals are likely to enjoy.

From an individual differences perspective, vocational interests and key personality traits, especially those related to Intellect and Lack of Worry/Emotionality in achievement contexts, were related to task preferences. Taken together with the preference modeling, however, one could reasonably conclude that many of those individuals who score high in Intellect, for example, may not necessarily be *attracted* to reading and writing tasks, but rather they might find such tasks less *aversive* than those individuals who are low in Intellect. Those who experience worry or negative emotions in achievement contexts are more attracted to games than those who do not report experiencing these emotional reactions. Enterprising and Conventional interests appear to be mostly related to heightened aversion to tasks with substantial cognitive demands (e.g., retirement planning, helping people complete IRS forms). It is possible that implicit negative wording of the instructions (i.e., participants were asked to indicate the minimum amount of money required in order to complete the task rather than the amount they would be willing to pay to do so) could have influenced this outcome, and further that this is why even some activities for which people commonly pay had positive disutility judgments (i.e., participants on average reported requiring some amount of money in order to participate). However, despite this wording of the instructions, 37.5% of tasks (9 out of 24) included in the preference modeling were rated on average “attractive”. Disutility judgments for typically enjoyable activities such as visiting an art museum (mean = 0.26, SD = 10.65) or attending a play (mean = 0.24, SD = 11.93) tended to have an average around zero (indicating a willingness to participate for free) and high variability. Because a systematic priming effect as described above would not explain high variability in these judgments (rather, it would tend to have an upward influence on all participants’ disutility judgments), we again suggest that individual differences are important for interpreting preference judgments.

These results further suggest that motivational trait and personality trait assessments provide an incomplete representation of individuals’ orientation toward mastery, learning, and other intellectual tasks. That is, most self-report assessments of these traits only ask about an individual’s affect, with respect to individual tasks or task families. They don’t assess what tasks the individual would do, without any other extrinsic rewards. Taken together, the preference data and trait correlations in the current study suggest that, *ceteris paribus*, most of those who had high scores on nAch, Intellect, and related traits would still prefer to play games over tasks that are more educational, such as watching TED Talks or reading books or news magazines.

In addition, the results of the study provide empirical evidence to support the notion that task attraction/aversion is not just a unidimensional function of the amount of mental effort demanded by the task or activity. Although some tasks with very high demands (writing SAT- type essays) or very low mental effort demands (watching security cameras) are nearly universally judged to be aversive, there are many tasks that have similar mental demands yet have lower judged aversion. Taking account of the other dimensions of the MDS space (e.g., games vs. chores and the additional degree of physical demands) may inform the researcher or practitioner about the anticipated level of approach/avoidance by potential employees or students. Ultimately, it may be that the optimal intensity of desired mental and physical effort on the part of the individual is, if not as important as the direction of interests, at least a factor that represents a meaningful and significant predictor of job/career aspirations and future job satisfaction.

The results of this study support the notion that researchers constructing or modifying theories of attention may find it beneficial to consider both individual differences in task preferences and the continuum of perceived boredom vs. fatigue as potentially important inputs to the prediction of subjective reactions to task performance over extended periods of time. A deeper understanding of these individual differences as explored in the current study may help to explain constructs less well explained in the effort literature, such as flow. Flow is defined as the subjective sense of concentration, environmental control, and reward experienced by an individual when performing a challenging task that draws on high levels of requisite skill (Csikszentmihályi, 1988; Nakamura and Csikszentmihályi, 2002). However, the processes by which this state is entered and experienced are not well understood (Westbrook & Braver, 2015) and would likely be enhanced by increased attention to individual differences in task preferences or perceived effort. Static theories or short-term experiments may provide a limited view of the dynamics of attentional effort available to an individual or the effort expended by that individual, when considered over longer periods of task engagement (e.g., see Ackerman, 2011; Arai, 1912; Thorndike, 1900).

Finally, this work suggests that researchers who presume that all study participants are equally challenged or motivated to perform tasks in the laboratory or the field, regardless of the kind of demands of the imposed tasks (e.g., perceptual, central processing, verbal, numerical, spatial, and so on), because they receive a particular level of compensation, may be overlooking a significant influence on effort expended toward task engagement or completion. Although there has been salient research on demand characteristics of psychological studies (e.g., Orne, 1962), much of this work may be both out of date and not representative of the kinds of tasks imposed by current studies. Study participants at many universities, for example, are presented with study descriptions that may or may not be concordant with individual differences in approach/avoidance patterns, and thus may influence the kinds of individuals who sign up for such studies—a phenomenon that has not received any substantive investigation. Such differences between individual preferences may also influence task persistence or fatigue effects, which in turn, may introduce variance in performance that is confounded between task and participant characteristics. Future research might investigate individual characteristics in preferences and salient personality/self-concept trait complexes as potentially influential covariates of performance in tasks as diverse as working memory or multitasking activities. As Underwood (1975) suggested, individual differences can serve as a useful “crucible” for constructing theories in experimental psychology.

## Data Availability

Data will be available on request to the first author.

## Abbreviations

AIC: Akaike information criterion
BIC: Bayesian information criterion
COGED: Cognitive effort discounting paradigm
IPIP: International Personality Item Pool
MDS: Multidimensional scaling
MTQ: Motivational Trait Questionnaire
nAch: Need for Achievement
TIE: Typical Intellectual Engagement
UNIACT: Unisex Edition of the American College Testing Interest Inventory

## Footnotes

1 Trait complexes (Ackerman & Heggestad, 1997) are groups of cross-domain (e.g., personality, interest, self-concept, ability) constructs that share common variance, indicated by correlations. They are a parsimonious way to represent a large number of traits that overlap and have synergistic effects, in terms of predicting individual differences in learning and performance (e.g., see Ackerman, Kanfer, & Beier, 2013).

2 When participants are faced with making hundreds of paired-comparison judgments, some participants may either shift their criteria partway through the task or are otherwise unreliable in their judgments (e.g., see Day, Deutscher, & Ryans, 1976). Based on prior experience with such judgments, a reliability threshold of *r* = .40 was set; 23 participants had reliabilities below this threshold. To evaluate whether unreliable judgments may have affected the results, MDS computations were performed for both the full set of participants and also only for those with above-threshold reliabilities. Results were highly consistent across solutions, with corresponding stimulus dimension loadings of *r* = .996 for Dimension I, *r* = .987 for Dimension II, and *r* = .985 for Dimension III. Given that there were no noticeable differences between solutions, we proceeded with the full sample for the final MDS solution.
